# Retraction: AFAP1-AS1 promotes epithelial-mesenchymal transition and tumorigenesis through Wnt/β-catenin signaling pathway in triple-negative breast cancer

**DOI:** 10.3389/fphar.2026.1782241

**Published:** 2026-01-16

**Authors:** Frontiers Editorial Office

The journal retracts the 16 November 2018 article cited above.

Following publication, concerns were raised regarding the integrity of the images in the published figures. The authors failed to provide a satisfactory explanation during the investigation, which was conducted in accordance with Frontiers' policies.

This retraction was approved by the Chief Editors of Frontiers in Pharmacology and the Chief Executive Editor of Frontiers. The authors received a communication regarding the retraction and had a chance to respond. This communication has been recorded by the publisher.

Frontiers would like to thank Kevin Patrick for contacting the journal regarding the published article.

